# Experiences with healthy eating among individuals with opioid dependence: insights from a dietary assessment

**DOI:** 10.1186/s40795-025-01106-1

**Published:** 2025-06-05

**Authors:** Mohammed Khalid Al-Shibli, Lars Thore Fadnes, Hege Berg Henriksen, Elaheh Javadi Arjmand, Lise Margrete Thomassen, Torgeir Gilje Lid, Siv-Elin Leirvåg Carlsen

**Affiliations:** 1 Bergen Addiction Research, Department of Addiction Medicine, Haukeland University Hospital, Bergen, Norway; 2 Department of Global Public Health and Primary Care, University of Bergen, Bergen, Norway; 3 Department of Nutrition, Institute of Basic Medical Sciences, University of Oslo, Oslo, Norway; 4 Centre for Alcohol and Drug Research, Stavanger University Hospital, Stavanger, Norway; 5 Department of Public Health, University of Stavanger, Stavanger, Norway; 6 Helse Bergen HF Haukeland Universitetssjukehus, Avdeling for Rusmedisin, Postboks 1400, Bergen, 5021 Norway

**Keywords:** Substance use disorders, Substance abuse, Opioid dependance, Opioid agonist therapy, Healthy diet, Interventions, Dietary feedback, Cross sectional study

## Abstract

**Background:**

People with substance use disorders often have unhealthy diets, including a limited intake of fruits and vegetables. Additionally, individuals with substance use disorders experience a significantly higher burden of physical and mental health conditions compared to the general population. Poor diets may contribute to this, but few studies have explored how these dietary habits could be improved. Therefore, our objective is to investigate the experiences with healthy eating among individuals with opioid dependence.

**Methods:**

We employed a qualitative design and recruited twelve patients undergoing opioid agonist therapy in Bergen. All participants were interviewed using a qualitative interview guide focused on experiences with healthy eating. Additionally, we conducted a dietary assessment using the DIGIKOST-FFQ tool, which was administered twice. Participants were then informed about how their diets aligned with the Norwegian dietary recommendations, and we interviewed them about their experiences with this information. Data analysis was carried out using systematic text condensation.

**Results:**

Our findings show that many participants recognized the potential health benefits of healthy eating and how these were negatively impacted by their substance use. Even if many participants recognized the potential health benefits of healthy eating and how these were negatively impacted by their substance use, they had mixed reactions to receiving personalized dietary feedback. They expressed a need for support from the healthcare system to help improving their diets.

**Conclusion:**

Our findings suggest that individuals with substance use disorders are interested in changing their diets but lack the skills to do so. While receiving personalized feedback may be effective for some, it would likely need to be combined with other interventions to improve their overall health.

**Supplementary Information:**

The online version contains supplementary material available at 10.1186/s40795-025-01106-1.

## Background

Substance use disorders (SUDs) encompass various conditions related to addiction to different substances, including opioids. Opioid dependence is associated with a significant burden of disease, including both physical and mental comorbidities, reduced quality of life, and decreased life expectancy, as well as nutritional risk factors [1–4]. Individuals with opioid dependence often consume high amounts of sugar, processed meats, and refined grain products, while their intake of fruits and vegetables tends to be low [5–8]. Nutritional deficiencies, such as low levels of certain vitamins, like vitamin A and folic acid, are also common in this group [9–12]. Not adhering to national dietary recommendations has also been shown to negatively affect psychological well-being, with several studies reporting higher rates of depression and other mental health disorders among people with SUDs [13, 14].

Several mechanisms have been proposed to explain why people with SUDs have unhealthy diets, including reduced interest in food [15], dental problems [16], and food insecurity [17]. Some studies also link struggles in meeting national dietary recommendations to impaired gastrointestinal health [18] and biochemical imbalances [19]. However, research on effective strategies to improve the diet of these individuals is limited. A systematic review of dietary interventions for people with illicit drug use identified only five relevant studies [20]. These interventions ranged from basic nutritional supplementation for individuals in outpatient clinics to counseling on healthy lifestyle habits for those in residential substance use treatment programs. While these studies reported significant improvements in both dietary habits and overall health outcomes, the specific results varied between studies, and follow-up periods were relatively short [21–25].

The Norwegian dietary guidelines aim to reduce non-communicable diseases and improve overall well-being in the general population [26]. The digital food frequency questionnaire (DIGIKOST-FFQ) assesses an individual’s diet in relation to the current national dietary recommendations [27–29]. Upon completing the questionnaire, participants receive a personalized report outlining how their diet al.igns with these recommendations, along with advice on how to make dietary adjustments. However, the use of this tool for individuals with SUD - mapping their dietary habits against national dietary guidelines and providing personalized recommendations - has not been previously studied.

This study aims to explore the experiences and perceptions of healthy eating among individuals with opioid dependence, as well as their experiences with dietary assessment using the DIGIKOST-FFQ application. The study will also examine barriers and facilitators to healthy eating in this population.

## Methods

### Study design

This is a qualitative study supported by descriptive quantitative data on participants dietary habits, linked to the ATLAS4LAR project. The ATLAS4LAR project aimed to identify interventions that could improve the physical and mental health of individuals with opioid dependence receiving opioid agonist therapy (OAT) [30].

The interview guide was developed collaboratively by researchers, research nurses, clinicians, and user representatives. It focused on barriers and facilitators to healthy eating, participants’ experiences with and interpretation of the DIGIKOST-FFQ application, as well as their adherence to dietary guidelines based on the individual reports from DIGIKOST-FFQ. A semi-structured qualitative design was chosen to allow flexibility in exploring both a priori and participant generated themes that were introduced. A translation of the interview guide is provided in the appendix 1.

### Setting and participants

The parent ATLAS4LAR project recruited people from OAT outpatient clinics in Bergen and Stavanger to a prospective cohort study with annual assessments from a source population of about 2,000 persons. All people receiving OAT from involved clinics receiving follow-up at least weekly were invited to the parent study and provided written consent. The participants of this study in Bergen were recruited purposively to this study, with consecutive assessment of characteristics aiming to represent different combinations of age, sex, and level of functioning (inversely correlated to frequency of clinical follow-up). Participants were recruited between November 2023 and January 2024 from three OAT clinics in the Bergen municipality.

In these clinics, a multidisciplinary team of nurses and social workers, supported by physicians and psychologists, provides follow-up care for patients. Patients receive OAT medication (e.g., methadone or buprenorphine) usually at least once a week and undergo an annual medical examination, along with other health-related follow-ups at the clinics. Descriptions of the participants are provided in Table 1. More detailed information about the setting and context of the clinics can be found in Fadnes et al. [31].

**Table 1:** Characteristics of the participants

**Gender**, *n*	
Male	9 (75%)
Female	3 (25%)
**Age**, mean (sd)	47 (7.1)
**BMI**, mean (sd)	23 (8.6)
**OAT medication**, n	
Methadone	8 (67%)
Buprenorphine	4 (33%)
**Education**, n	
Not finished basic education	2 (18%)
Finished basic education	8 (73%)
High school	1 (9%)
**Substance use** Illicit opioids, n	
None	9 (82%)
< 3 times per week	2 (18%)
Alcohol, n	
None	4 (36%)
< 3 times per week	6 (55%)
≥ 3 times per week	1 (9%)
Stimulants, n	
None	9 (82%)
< 3 times per week	2 (18%)
Cannabis, n	
None	2 (18%)
< 3 times per week	4 (36%)
≥ 3 times per week	5 (46%)
Tobacco, n	
Daily	12 (100%)
Total amount of participants	12

### Data collection

After receiving information and signing the consent form, participants’ dietary intake was assessed using the DIGIKOST-FFQ. Following this, individual interviews were conducted by research nurses which had completed specific training in qualitative interviewing techniques. There were three female research nurses that each provided 3, 4 and 5 dietary assessment followed by semi-structured one-to-one interviews. We originally conducted nine interviews, and were starting to see thematic saturation (i.e., when identified themes have emerged before and added interviews do not add more to the information power [32]). We then added three more interviews to see if there were any major changes in themes, which we did not see, and therefore stopped further interviewing. All participants had previously met the research nurses at least once as part of their involvement in the ATLAS4LAR study. The interviews were audio-recorded using a Sony ICD-PX470 dictaphone and uploaded to a secure research server (managed by Helse Bergen, stored as mp3-files). All interviews were then transcribed verbatim by the first-author and stored on the same secure server. Transcriptions were de-identified and given ID-numbers, based on the order in which the interviews were conducted.

The DIGIKOST-FFQ was used to estimate dietary intake and monitor adherence to the Norwegian dietary guidelines. Completing the DIGIKOST-FFQ took approximately 20 min. The questionnaire includes:

- A total of 78 questions about food items (e.g., grams per day of fruits and vegetables, whole grains, nuts, fish, meat, dairy products, major sources of added sugars including beverages, and fat sources).
- Seven questions about physical activity (minutes per week), sedentary behavior, and sleep (hours per day).
- Eight questions about tobacco use.
- Ten questions about body weight and demographic data.

The DIGIKOST-FFQ automatically generates a digital report on respondents’ adherence to the dietary guidelines, offering specific and personalized advice on how to meet the recommendations. DIGIKOST-FFQ uses a software platform and the responses are directly transferred to the secure server for sensitive data. Data on participants’ intake of fruits, vegetables, whole grains, fish, red meat, high-fat foods, and high-sugar foods were extracted and compared to the recommended levels.

### Data analysis

**Fig. 1:**
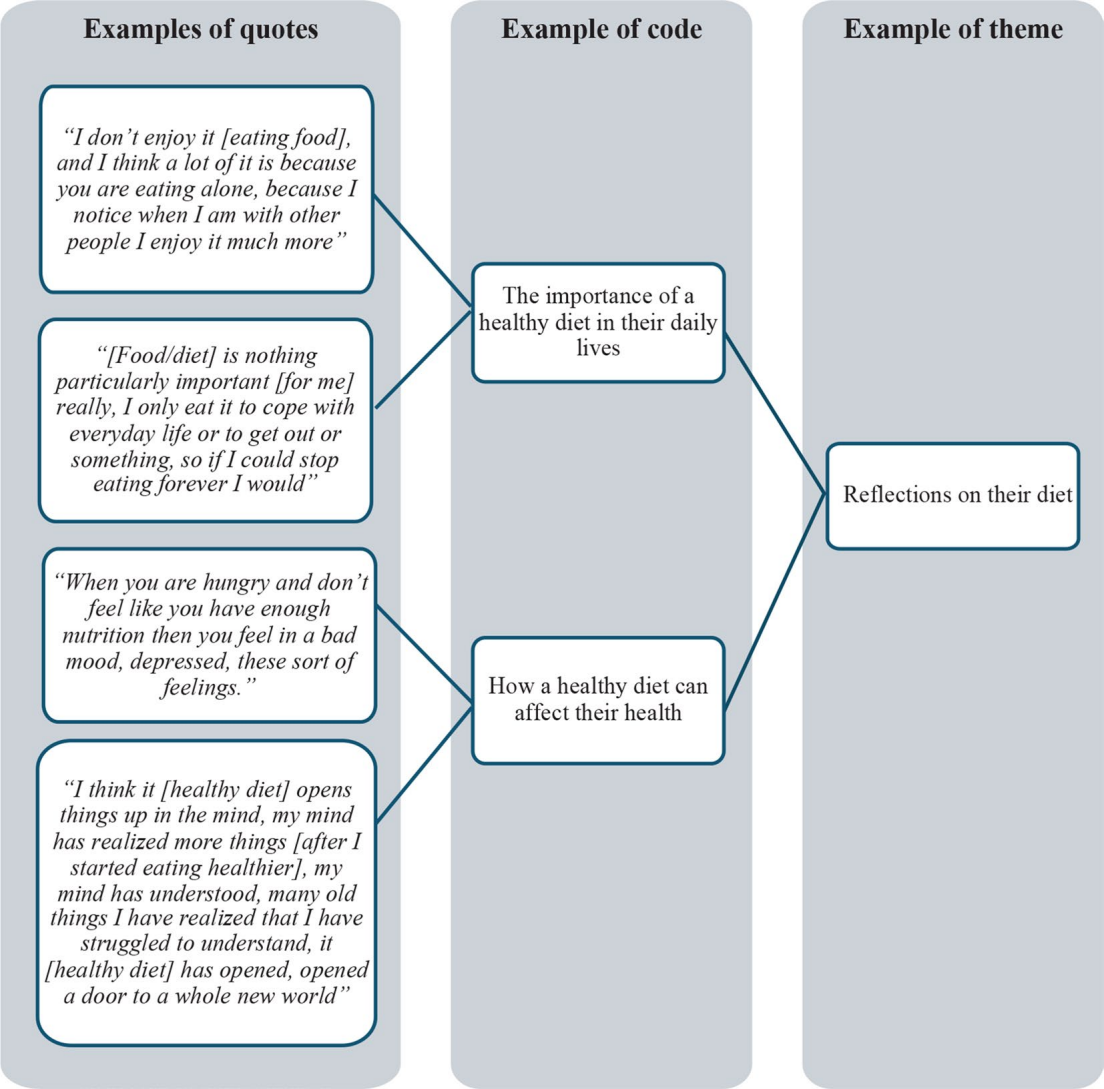
An illustration of the analysis process. After reading multiple times through the transcripts, the authors highlighted the most extensive and representative quotes which were coded using NVIVO 12, and then used to indentify the main themes of the study

**Fig. 2:**
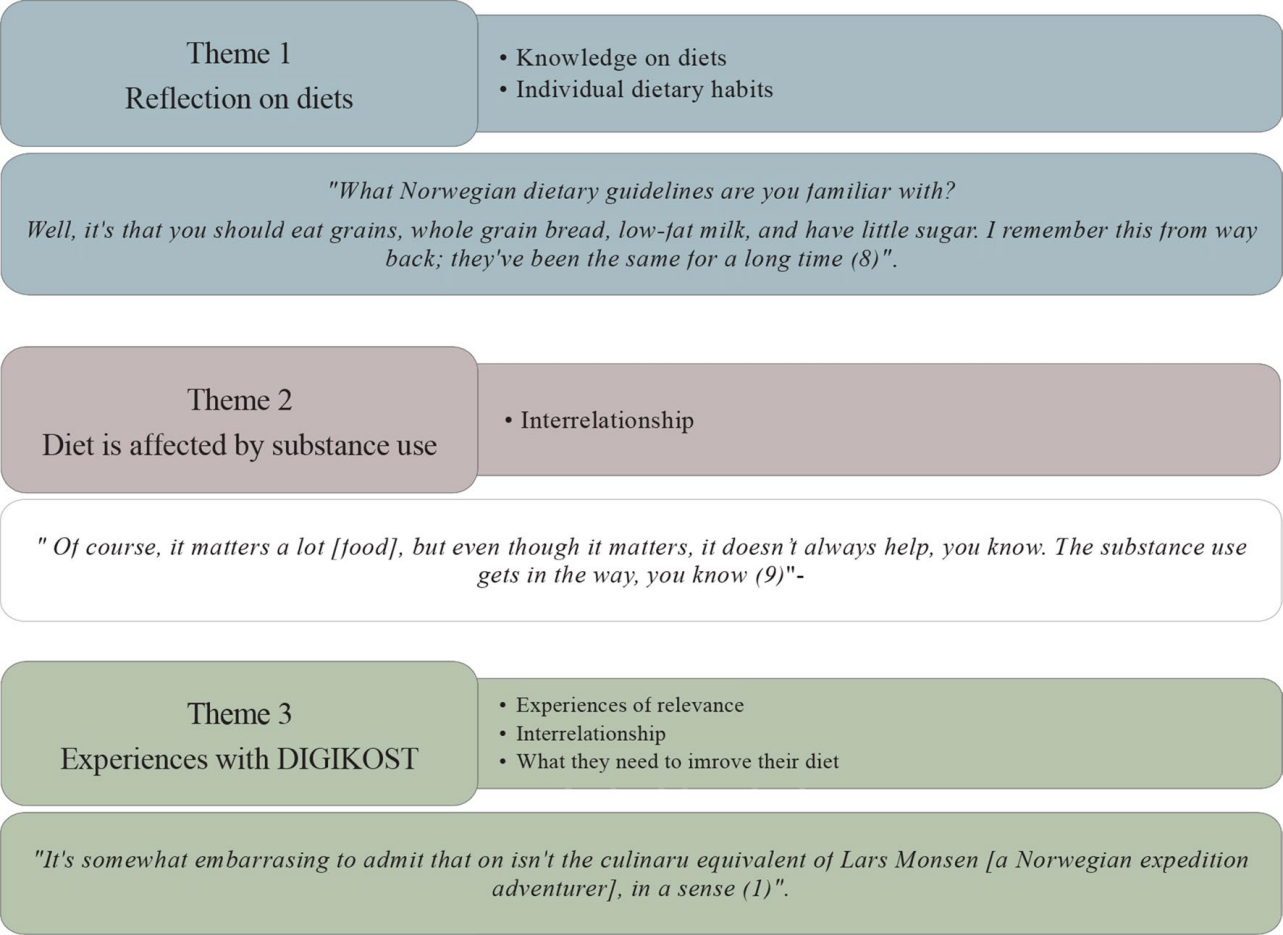
Process of identifying themes and sub-themes. Main themes were identified and we divided them into sub-themes after re-reading the transcripts. We then identified a quote which best summed up the view of the participants. Number in the paranthesis after each quote is the number which each participant was assigned

The researchers employed thematic analysis to identify, analyze, and report themes. A bottom-up approach was utilized, and the qualitative methodology was chosen to expand on quantitative studies establishing insufficient diets among patients receiving OAT [4, 8]. The analysis followed the four steps of systematic text condensation, a strategy for thematic analyis designed to facilitate a process of intersubjectivity and reflexivity while maintaining methodological quality, and thus well suited for less experienced researchers [33, 34]. In the first step, two authors (MKA and SELC) read through the transcripts to familiarize themselves with the material. In the second step, they re-read the transcripts and separately coded the interviews into initial themes. The researchers then compared and discussed their individual codes, removing or recoding themes as needed. To ensure comprehensive coverage and address any potential oversights, the codes and themes were reviewed and discussed with the coauthors. In the third step, key aspects of each code group were identified, and a condensation was performed. The final step involved creating an analytic text based on the condensed data to be included in the final article. Fig. 1 provides an illustration of the analysis process. A final codebook was applied to the data, and NVIVO 14 [35] was used to generate the primary themes and subthemes. This organization into themes and subthemes is summarized in Fig. 2.

### Ethics

The study was approved in accordance with the Declaration of Helsinki, by the Regional Ethical Committee south-east, no. 155,386/ REK, dated 23.09.2020/05.04.2021.

## Results

The participants in this study included nine males and three females - with a mean age of 47 years (standard deviation of 7.1 years). All participants had stable living conditions, defined as either owning or renting their own place of residence, but none had a regular income. Two participants had not completed basic education, while the remaining had completed either basic or higher levels of education (Table 1). The majority were receiving methadone as their OAT medication (67%). The interviews were audio-recorded with a dictaphone and lasted between 11 and 29 min.

### Reflections on their diet

#### Perceptions of a healthy diet

When participants were asked to recall what they believed a healthy diet consisted of, most could accurately describe the need to eat fish, fruits, vegetables, and grains while limiting processed foods. However, their knowledge of the dietary guidelines varied. Some participants demonstrated a good understanding of the guidelines, while others reported no familiarity with them. Many, however, mentioned having learned about the “dietary circle” or other dietary recommendations, either during their younger years or while undergoing treatment for substance use.

*A circle divided like a pizza*,* and eating healthy*,* like grains and meat*,* was a big part of it. A small piece of that pizza was for sugar*,* sweets*,* and soft drinks. I don’t remember exactly how big*,* but I know what you should eat the most of.– Participant nr.1 (female)*

Most participants acknowledged that they were not adhering to many of the dietary recommendations. While they believed they should be eating better, they still often opted for unhealthy food choices. The main reasons for this discrepancy were the perceived high cost of healthy foods and a lack of motivation to cook, often due to loneliness. Despite this, some participants perceived their diet as sufficient, believing that national dietary recommendations did not apply to them because their eating habits were significantly different from the norm. More than half of the participants stated that food was not important to them, serving only to provide energy and nourishment for survival. The increased focus on healthy food in the society, might make the pursuit of food and staying “healthy” seem like a burden. Other reasons cited included social considerations, such as eating with others, and avoiding the appearance of being excessively thin.

*[Food] is nothing particularly important [for me] really. I only eat it to cope with everyday life or to get out of something. So*,* if I could stop eating forever [without health consequences]*,* I would.– Participant nr.11 (male)*

### How a healthy diet affects their health

Despite acknowledging that they did not prioritize their diet, many participants still emphasized its importance for both physical and mental well-being. Some admitted that their unhealthy eating habits were harming their health. They observed that the significance of maintaining a healthy diet could vary considerably depending on their overall well-being. When facing challenges in other areas of life, they felt less motivated to eat healthily. Conversely, when their circumstances improved and they experienced greater mental clarity, their motivation to adopt healthier eating habits increased, which in turn boosted their self-esteem. Some participants also recalled periods in their lives when they prioritized healthy eating.

*When I started eating healthy*,* I noticed it on my whole body*,* hair*,* mood*,* skin*,* teeth*,* everything*,* and on how I behaved towards others also. When you don’t get food*,* you’re sad and cranky*,* but when you know you’re getting food*,* your mood changes.– Participant nr.4 (female)*

Several participants reported prioritizing healthy eating during earlier periods of their lives, such as when they lived with others, held jobs, engaged in sports, or underwent treatment. During those times, they were more conscious of their diet and felt successful in maintaining healthy eating habits. However, these periods were short-lived, as their motivation declined and their focus on healthy eating was overtaken by the pursuit of drugs during more challenging times.

*When I was in contact with other people [I used to eat healthier]*,* now when I started isolating myself staying at home alone*,* I started eating badly*,* but that was different when I had friends visiting.– Participant nr.5 (male)*

### Diet is affected by substance use

When asked if they thought a healthy diet could influence their drug cravings, some participants reported using fewer drugs when they ate healthily, while many others perceived no connection between the two. A few mentioned the positive feelings they experienced from eating food, especially sweet foods, and how this feeling could mimic the effects of drug use. Negative thoughts about not eating healthily could impact their self-image, leading to increased drug cravings to cope with these negative emotions, as one participant explained:

*It’s easy to take drugs to drown them [negative thoughts about not eating sufficiently] away. Thoughts like you don’t get what you need*,* or that I don’t have energy when I sleep badly. And I hardly sleep*,* so this is all quite a disorganized mixture.– Participant nr.2 (male)*

Some participants emphasized that they did not necessarily believe food intake influenced their drug cravings. Instead, they felt that their drug use affected their food choices, with drugs impacting their appetite and energy levels. Specifically, some participants mentioned using amphetamines to control weight gain, as noted by one participant:

*If you have taken amphetamines*,* for example*,* you don’t get hungry in the same way. You feel nauseous*,* and it becomes difficult to eat. So*,* it is more that the drugs make it harder to eat. It is not the diet that makes me want to use more drugs.– Participant nr.1 (female)*

Heroin use was also reported to influence participants’ intake of food, making them them less interested in eating, and they could end up not even eating at all.

*(…) but let’s say that you have taken heroin*,* then it is easy for you to just lie on the sofa instead of bothering to cook dinner or go out to buy something.– Participant nr.1 (female)*

One participant noted that young people with SUD were particularly at risk of eating unhealthily because they spent all their money on drugs, neglecting grocery shopping and leading to inadequate food intake. Some participants also mentioned that during periods of substance use, their apartments were often in a state of chaos, and their kitchens were not suitable for food preparation due to poor hygiene conditions.

### Eating as reported through DIGIKOST-FFQ and alignment with recommendations

**Fig. 3:**
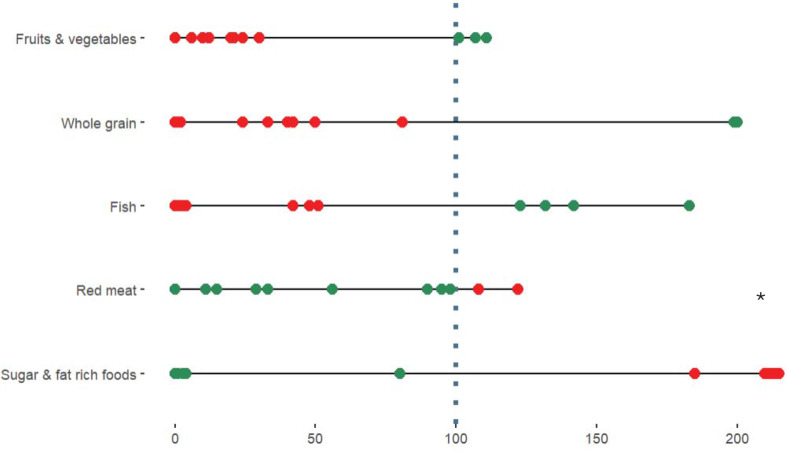
Adherence to the dietary guidelines (%). Adherence to the Norwegian dietary guidelines for five food groups are presented (*n* = 12). The red dots represent participants that have a lower/higher intake than the recommended intake levels, while green dots represent participants that have an intake level within the recommended levels. * = outliers in the range 320–1495%

An overview of participants’ i ntake of the main food groups and adherence to the Norwegian Diet Index [36], based on the DIGIKOST-FFQ, is shown in Fig. 3. Three of the 12 participants adhered to the dietary guidelines for fruits and vegetables, consuming more than 500 g per day. Two participants had a whole grain intake above the recommended level of 90 g per day, and four participants reported a fish intake of over 300 g per week. All but two participants had a meat consumption of less than 500 g per week. The results for sugar- and fat-rich foods varied significantly between participants. While four participants reported an intake of sugar- and fat-rich foods below 20 g per day, six participants had intake levels several times higher than the recommended amounts. None adhered to all of the dietary recommendations.

#### Participants perceptions of the DIGIKOST-FFQ tool

Despite many of their results not meeting the dietary guidelines, most participants reported positive experiences with the DIGIKOST-FFQ. Nearly all found the questionnaire easy to use and the questions understandable. Another positive aspect was receiving a printed report, which helped them remember the recommendations if they forgot. Some participants also appreciated the personalized feedback on how to improve their diet, as it showed that small changes could make a significant difference. Receiving positive feedback was enjoyable for some and contributed to boosting their self-esteem.

*[It is good getting feedback] that we eat what we are supposed to eat*,* and just knowing that you’re not completely losers*,* because there’s a lot of stigmas*,* and a lot of negativities with the people I talk to*,* so hearing positive things is good.– Participant nr.4 (female)*

Some participants found the entire dietary screening to be superficial and did not pay much attention to the feedback. They reported that they were already aware of their diet’s shortcomings, and being reminded of this was not seen as helpful in improving their eating habits. Others found the negative feedback in the report to be embarrassing, which negatively impacted their mental health and self-confidence.

*I just get more depressed [because of the negative feedback]*,* think there’s just one more thing I can’t do. I can’t do anything. I feel*,* maybe we should now sit in with the psychologist.– Participant nr.11 (male)*

On the other hand, several participants expressed that the DIGIKOST-FFQ report increased their awareness of their diet, potentially making them more mindful when shopping for food. However, many also noted that while the report was a good first step, it was unlikely to lead to long-term changes in their eating habits. Participants emphasized that to improve their diet, they would first need support in other areas of their lives, particularly regarding their financial situation and establishing more structure in their daily routines. Addressing these issues would better enable them to prioritize their diet.

#### Tools and actions to facilitate healthier eating

Participants were asked to reflect on tools and measures that could help them eat healthier. While two participants could not imagine any specific measures, most expressed a desire for some form of support. One type of support mentioned by participants was food distribution. Many were aware of where and which institutions who distributed nutritious food, whether it was breakfast/lunch or dinner, and some took advantage of this occasionally. However, many emphasized the need for increased knowledge, not only about what they should eat but also regarding practical aspects such as meal preparation and cooking techniques.

*[I could need someone to] help me buy fruit and vegetables and help me mix it so it tastes good. Because we have had vegetables in the fridge that have rotted because we did not know what to do with it.– Participant nr.8 (male)*

Other suggestions for improving their diet included having someone to live with, consuming nutrient drinks, receiving financial support, and having the opportunity to participate in scheduled group physical exercise to help increase appetite.

*In order to eat more vegetables*,* I usually always make myself a wok*,* but why bother when I’m alone*,* right… I’ve always been used to living with [other people].– Participant nr.12 (male)*

## Discussion

The aim of this study was to understand how healthy eating is perceived among people with opioid dependence and to explore their experiences with dietary assessment and feedback on adherence to national dietary recommendations. Most participants could to some degree describe what constitutes a healthy diet and identified areas where their diet could be improved. Nearly all participants recognized the positive health effects of maintaining a healthy diet and acknowledged that neglecting their diet negatively impacted their overall health. Although many participants had sufficient knowledge of what they should eat to improve their health, many requested more practical guidance on how to prepare food in a healthy way.

Despite the perception that people with SUD are not interested in improving their diet, our findings suggest that many of these patients are motivated to improve both their diets and overall quality of life. This motivation is particularly strong in social settings, such as eating with others or in environments where food is readily available, or where there are opportunities to cook together. Many participants emphasized the importance of social factors in improving their diets [37]. An increased focus on healthy eating in outpatient clinics could improve their diets, for example, by providing nutritionous foods or drinks [38]. This would also help participants who reported that healthy food options were too expensive compared to unhealthy ones. A study done in Vermont showed promising results, where patients could pick up healthy foods for free at a methadone maintenance clinic, providing insights as to what may be an effective measure against food insecurity [39]. This type of arrangement was requested by some of the participants in our study. Charities and organizations that offer free meals, should also make their services more visible, helping OAT patients become more aware of and better able to utilize them [40].

Our study shows that participants valued healthy eating more during periods when life was going well, a finding supported by a study from England [41]. However, during more challenging times, their diet was often one of the first things to be neglected. Participants also reported that substance use affected their appetite and energy levels, leading them to choose unhealthy foods. Our findings also suggest that maintaining a healthy diet could boost self-esteem, making substance use less appealing. Trials conducted among individuals with mental health disorders have indicated that healthy eating can improve mental health [42]. Given the strong connections between substance use disorders and mental health symptoms [43], it is reasonable to assume that healthy eating could also contribute positively to reducing substance use [44].

Furthermore, a diet high in fruits and vegetables and low in sugary foods and processed meats could lower the risk of somatic morbidities and all-cause mortality [45–47], which are prevalent within this population [4]. Due to the compulsive nature of substance use, individuals may substitute substances with unhealthy foods, such as those with a sweet taste, known as an addictive switch [48]. This addicitive switch can cause excessive weight gain, which may become even more severe in patients receiving methadone as their OAT medication, which eight out of the twelve participants in our study did [49, 50]. Since this can lead to adverse health outcomes like obesity, it is essential to incorporate a nutritional component into OAT [51].

Assessing the nutritional status of individuals receiving OAT helps identify the risk of malnutrition and associated comorbidities. This study specifically explored the use of the DIGIKOST-FFQ as a tool to assess patients’ diets and provide personalized feedback. Some participants supported integrating this type of digital screening tool into OAT clinics, as it could encourage them to focus on healthy eating. However, others felt that this tool would not help them eat more healthily, either because they did not enjoy using it or believed they needed support in other areas before they could improve their diet. Our findings suggest that participants who did not appreciate having their diet assessed with the DIGIKOST-FFQ, were often those who reported that food held little significance in their daily lives. This suggests that careful consideration is needed regarding which patients are screened with such tools, as it may benefit some while being less helpful to others.

In this group of participants with SUD, we found a low intake of whole grains, fruits, and vegetables, consistent with previous findings [5]. One explanation for the low intake of fruits and vegetables could be the poor dental health reported by this patient group, which makes many fruits and vegetables difficult to chew [16]. One way of combatting this, could be by providing fruit smoothies, which have been shown to be a feasible and well-received intervention by patients [38]. Poor dental health could also contribute to the finding that all but two participants had a red meat intake within the recommended levels, as many meat products can also be difficult to chew. Another possible reason for the low intake of fruits and grains, may be the dysregulated gut microbiome that is thought to frequent in this population, causing discomfort upon consuming certain types of foods [52, 53].

### Strengths and limitations

To our knowledge, this study is among the first to use a digital screening tool designed to provide personalized nutritional feedback to patients receiving OAT, offering new insights into how it was perceived. The interviews were semi-structured and not intended to delve deeply into all topics, which may have limited the exploration of some potentially interesting themes. While some degree of socially desirable reporting cannot be excluded, significant bias is unlikely, as the interviews and nutritional assessments using DIGIKOST-FFQ had no impact on their current OAT treatment, and the interviews were conducted by research nurses with no involvement in the development of the DIGIKOST-FFQ. Participants were also informed that their answers would remain anonymous. In terms of transferability, our results are most relevant to individuals with opioid use disorders who are receiving regular follow-up care from the healthcare system.

Some of the participants were more articulated than others, and therefore not all participants are represented with quotes. Some respondents provided brief answers, while others gave more elaborate responses with numerous digressions, making it cumbersome to extract the meaning if providing these quotes. However, all statements from the participants were taken into consideration in the synopsis of the differens themes. The interviewers were highly experienced and had extensive knowledge of and familiarity with the patient population. This may have contributed to fewer follow-up questions being asked, as they implicitly understood what the participants were expressing. This prior understanding was often not challenged, which might have been the case if the interviews had been conducted by people without this background knowledge. The concept of saturation can be challenging [32], as new nuances often emerge with additional interviews even though there might not be new themes. Our assessment was that by the ninth interview, we observed repetitive patterns in the data, and after three additional interviews, no new themes were introduced. Due to the short interview guide the potential for new themes to emerge, were a bit limitied. This is a limitation with our study.

## Conclusions

These findings suggest that patients receiving OAT have low adherence to dietary recommendations but nevertheless that many express a desire to improve their diets and, consequently, their overall health. While they possess some knowledge about healthy eating and what they should consume, they often lack the ability to apply this in their daily lives. A dietary screening tool, such as the DIGIKOST-FFQ, may be useful in assessing dietary intake and adherence to national dietary recommendations for patients who are motivated to improve their diet. However, many called for more support for improving their diets.

## Electronic supplementary material

Below is the link to the electronic supplementary material.

Supplementary Material 1

## Data Availability

No datasets were generated or analysed during the current study.

## Abbreviations

DIGIKOST-FFQ: Digital food frequency questionnaire
OAT: Opioid agonist treatment
OUD: Opioid use disorder
SUD: Substance use disorder(s)

## References

[1] Gryczynski J, Schwartz RP, O’Grady KE, Restivo L, Mitchell SG, Jaffe JH. Understanding patterns of High-Cost health care use across different substance user groups. Health Aff (Millwood). 2016;35(1):12–9. 26733696 10.1377/hlthaff.2015.0618 PMC4936480

[2] Lewer D, Jones NR, Hickman M, Nielsen S, Degenhardt L. Life expectancy of people who are dependent on opioids: A cohort study in new South wales, Australia. J Psychiatr Res. 2020;130:435–40. 32905957 10.1016/j.jpsychires.2020.08.013

[3] Rehm J, Manthey J, Shield KD, Ferreira-Borges C. Trends in substance use and in the attributable burden of disease and mortality in the WHO European region, 2010-16. Eur J Public Health. 2019;29(4):723–8. 31008515 10.1093/eurpub/ckz064

[4] Nabipour S, Ayu Said M, Hussain Habil M. Burden and nutritional deficiencies in opiate addiction- systematic review Article. Iran J Public Health. 2014;43(8):1022–32. 25927032 PMC4411899

[5] Saeland M, Haugen M, Eriksen FL, Wandel M, Smehaugen A, Böhmer T, et al. High sugar consumption and poor nutrient intake among drug addicts in oslo, Norway. Br J Nutr. 2011;105(4):618–24. 20880416 10.1017/S0007114510003971

[6] Saeland M, Haugen M, Eriksen FL, Smehaugen A, Wandel M, Böhmer T, et al. Living as a drug addict in oslo, Norway–a study focusing on nutrition and health. Public Health Nutr. 2009;12(5):630–6. 18549520 10.1017/S1368980008002553

[7] Nolan LJ, Scagnelli LM. Preference for sweet foods and higher body mass index in patients being treated in long-term methadone maintenance. Subst Use Misuse. 2007;42(10):1555–66. 17918026 10.1080/10826080701517727

[8] Morabia A, Fabre J, Chee E, Zeger S, Orsat E, Robert A. Diet and opiate addiction: a quantitative assessment of the diet of non-institutionalized opiate addicts. Br J Addict. 1989;84(2):173–80. 2720181 10.1111/j.1360-0443.1989.tb00566.x

[9] Bemanian M, Vold JH, Chowdhury R, Aas CF, Gjestad R, Johansson KA et al. Folate status as a nutritional Indicator among people with substance use disorder; A prospective cohort study in Norway. Int J Environ Res Public Health. 2022;19(9). 10.3390/ijerph19095754 PMC9099634 35565159

[10] Ross LJ, Wilson M, Banks M, Rezannah F, Daglish M. Prevalence of malnutrition and nutritional risk factors in patients undergoing alcohol and drug treatment. Nutrition. 2012;28(7–8):738–43. 22356728 10.1016/j.nut.2011.11.003

[11] el-Nakah A, Frank O, Louria DB, Quinones MA, Baker H. A vitamin profile of heroin addiction. Am J Public Health. 1979;69(10):1058–60. 484761 10.2105/ajph.69.10.1058 PMC1619165

[12] Yazici AB, Akcay Ciner O, Yazici E, Cilli AS, Dogan B, Erol A. Comparison of vitamin B12, vitamin D and folic acid blood levels in patients with schizophrenia, drug addiction and controls. J Clin Neurosci. 2019;65:11–6. 31076249 10.1016/j.jocn.2019.04.031

[13] Aas CF, Vold JH, Gjestad R, Skurtveit S, Lim AG, Gjerde KV, et al. Substance use and symptoms of mental health disorders: a prospective cohort of patients with severe substance use disorders in Norway. Subst Abuse Treat Prev Policy. 2021;16(1):20. 33639969 10.1186/s13011-021-00354-1 PMC7912462

[14] Armoon B, Fleury MJ, Bayat AH, Bayani A, Mohammadi R, Griffiths MD. Quality of life and its correlated factors among patients with substance use disorders: a systematic review and meta-analysis. Arch Public Health. 2022;80(1):179. 35927697 10.1186/s13690-022-00940-0 PMC9351239

[15] Volkow ND, Wang GJ, Baler RD. Reward, dopamine and the control of food intake: implications for obesity. Trends Cogn Sci. 2011;15(1):37–46. 21109477 10.1016/j.tics.2010.11.001 PMC3124340

[16] Teoh L, Moses G, McCullough MJ. Oral manifestations of illicit drug use. Aust Dent J. 2019;64(3):213–22. 31309583 10.1111/adj.12709

[17] Davison KM, Holloway C, Gondara L, Hatcher AS. Independent associations and effect modification between lifetime substance use and recent mood disorder diagnosis with household food insecurity. PLoS ONE. 2018;13(1):e0191072. 29360862 10.1371/journal.pone.0191072 PMC5779657

[18] Wiss DA. A biopsychosocial overview of the opioid crisis: considering nutrition and Gastrointestinal health. Front Public Health. 2019;7:193. 31338359 10.3389/fpubh.2019.00193 PMC6629782

[19] Mannelli P, Patkar A, Rozen S, Matson W, Krishnan R, Kaddurah-Daouk R. Opioid use affects antioxidant activity and purine metabolism: preliminary results. Hum Psychopharmacol. 2009;24(8):666–75. 19760630 10.1002/hup.1068 PMC3183957

[20] Whatnall MC, Skinner J, Pursey K, Brain K, Collins R, Hutchesson MJ, et al. Efficacy of dietary interventions in individuals with substance use disorders for illicit substances or illicit use of pharmaceutical substances: A systematic review. J Hum Nutr Diet. 2021;34(6):981–93. 33650747 10.1111/jhn.12871

[21] Juel A, Kristiansen CB, Madsen NJ, Munk-Jørgensen P, Hjorth P. Interventions to improve lifestyle and quality-of-life in patients with concurrent mental illness and substance use. Nord J Psychiatry. 2017;71(3):197–204. 27834103 10.1080/08039488.2016.1251610

[22] Buydens-Branchey L, Branchey M, Hibbeln JR. Associations between increases in plasma n-3 polyunsaturated fatty acids following supplementation and decreases in anger and anxiety in substance abusers. Prog Neuropsychopharmacol Biol Psychiatry. 2008;32(2):568–75. 18060675 10.1016/j.pnpbp.2007.10.020 PMC2275606

[23] Tremain D, Freund M, Wye P, Bowman J, Wolfenden L, Dunlop A, et al. Providing routine chronic disease preventive care in community substance use services: a pilot study of a multistrategic clinical practice change intervention. BMJ Open. 2018;8(8):e020042. 30121589 10.1136/bmjopen-2017-020042 PMC6104796

[24] Cowan JA, Devine CM. Diet and body composition outcomes of an environmental and educational intervention among men in treatment for substance addiction. J Nutr Educ Behav. 2013;45(2):154–8. 22633178 10.1016/j.jneb.2011.10.011 PMC3430793

[25] Kelly PJ, Baker AL, Townsend CJ, Deane FP, Callister R, Collins CE, et al. Healthy recovery: A pilot study of a smoking and other health behavior change intervention for people attending residential alcohol and other substance dependence treatment. J Dual Diagn. 2019;15(3):207–16. 31122158 10.1080/15504263.2019.1612537

[26] Helsedirektoratet. Kostrådene H. 2024; [Available from https://www.helsenorge.no/kosthold-og-ernaring/kostrad/helsedirektoratets-kostrad/?gad_source=1&gclid=CjwKCAjwm_SzBhAsEiwAXE2CvxFaECe2orqTlAO5fjIqRbbijJzxWIIrJj3Nylcsf-rdRqoe4QLLIxoCfOIQAvD_BwE

[27] Henriksen HB, Knudsen MD, Carlsen MH, Hjartåker A, Blomhoff R. A short digital food frequency questionnaire (DIGIKOST-FFQ) assessing dietary intake and other lifestyle factors among norwegians: qualitative evaluation with focus group interviews and usability testing. JMIR Form Res. 2022;6(11):e35933. 36346647 10.2196/35933 PMC9682459

[28] Knudsen MD, Carlsen MH, Hjartåker A, Blomhoff R, Henriksen HB. Reproducibility and comparison of a digital food frequency questionnaire (DIGIKOST-FFQ) assessing adherence to National diet and lifestyle recommendations. Food Nutr Res. 2024;68. 10.29219/fnr.v68.10366 PMC11375443 39239454

[29] Henriksen HB, Knudsen MD, Hjartåker A, Blomhoff R, Carlsen MH. Digital food frequency questionnaire assessing adherence to the Norwegian food-Based dietary guidelines and other National lifestyle recommendations: instrument validation study. J Med Internet Res. 2024;26:e53442. 38687986 10.2196/53442 PMC11094607

[30] Bergen H. ATLAS4LAR: Kartlegging og behandling av lungesykdom i legemiddelassistert behandling [ATLAS4LAR: Mapping and treatment of pulmonary disease in OAT]: Helse Bergen. 2022; [Available from: https://www.helse-bergen.no/avdelinger/rusmedisin/rusmedisin-seksjon-forsking/bar/atlas4lar-kartlegging-og-behandling-av-lungesykdom-i-legemiddelassistert-behandling

[31] Fadnes LT, Aas CF, Vold JH, Leiva RA, Ohldieck C, Chalabianloo F, et al. Integrated treatment of hepatitis C virus infection among people who inject drugs: A multicenter randomized controlled trial (INTRO-HCV). PLoS Med. 2021;18(6):e1003653. 34061883 10.1371/journal.pmed.1003653 PMC8205181

[32] Malterud K, Siersma VD, Guassora AD. Sample size in qualitative interview studies: guided by information power. Qual Health Res. 2016;26(13):1753–60. 10.1177/1049732315617444. 26613970 10.1177/1049732315617444

[33] Malterud K. Kvalitative Metoder i Medisinsk forskning: En Innføring [Qualitative methods in medical research: an introduction]. Oslo: Universitetsforl.; 2011.

[34] Malterud K. Systematic text condensation: a strategy for qualitative analysis. Scand J Public Health. 2012;40(8):795–805. 23221918 10.1177/1403494812465030

[35] Lumivero. NVivo 14 - Leading qualitative data analysis software with AI solution: Lumivero. 2024; [Available from: https://lumivero.com/products/nvivo/

[36] Henriksen HB, Berg HB, Andersen LF, Weedon-Fekjær H, Blomhoff R. Development of the Norwegian diet index and the Norwegian lifestyle index and evaluation in a National survey. Food Nutr Res. 2023;67. 10.29219/fnr.v67.9217 PMC10552792 37808205

[37] Mahboub N, Rizk R, Karavetian M, de Vries N. Nutritional status and eating habits of people who use drugs and/or are undergoing treatment for recovery: a narrative review. Nutr Rev. 2021;79(6):627–35. 10.1093/nutrit/nuaa095 PMC8114851 32974658

[38] Furulund E, Druckrey-Fiskaaen KT, Carlsen S-EL, Madebo T, Fadnes LT, Lid TG. Healthy eating among people on opioid agonist therapy: a qualitative study of patients’ experiences and perspectives. BMC Nutr. 2024;10(1):70. 38705977 10.1186/s40795-024-00880-8 PMC11071228

[39] Sigmon SC. Additive burdens of malnutrition, poverty, and substance abuse. Lancet. 2016;388(10054):1879–80. 27751393 10.1016/S0140-6736(16)31814-1 PMC5514411

[40] Naumann RB, Frank M, Shanahan ME, Reyes HLM, Ammerman AS, Corbie G, et al. State supplemental nutrition assistance program policies and substance use rates. Am J Prev Med. 2024;66(3):526–33. 37918458 10.1016/j.amepre.2023.10.019 PMC12930790

[41] Neale J, Nettleton S, Pickering L, Fischer J. Eating patterns among heroin users: a qualitative study with implications for nutritional interventions. Addiction. 2012;107(3):635–41. 21933297 10.1111/j.1360-0443.2011.03660.x

[42] Opie RS, Itsiopoulos C, Parletta N, Sanchez-Villegas A, Akbaraly TN, Ruusunen A, et al. Dietary recommendations for the prevention of depression. Nutr Neurosci. 2017;20(3):161–71. 26317148 10.1179/1476830515Y.0000000043

[43] Burdzovic Andreas J, Lauritzen G, Nordfjaern T. Co-occurrence between mental distress and poly-drug use: a ten year prospective study of patients from substance abuse treatment. Addict Behav. 2015;48:71–8. 26004857 10.1016/j.addbeh.2015.05.001

[44] Jeynes KD, Gibson EL. The importance of nutrition in aiding recovery from substance use disorders: A review. Drug Alcohol Depend. 2017;179:229–39. 28806640 10.1016/j.drugalcdep.2017.07.006

[45] Schwingshackl L, Schwedhelm C, Hoffmann G, Lampousi AM, Knüppel S, Iqbal K, et al. Food groups and risk of all-cause mortality: a systematic review and meta-analysis of prospective studies. Am J Clin Nutr. 2017;105(6):1462–73. 28446499 10.3945/ajcn.117.153148

[46] Fadnes LT, Javadi Arjmand E, Økland JM, Celis-Morales C, Livingstone KM, Balakrishna R, et al. Life expectancy gains from dietary modifications: a comparative modeling study in 7 countries. Am J Clin Nutr. 2024;120(1):170–7. 38692410 10.1016/j.ajcnut.2024.04.028

[47] Aune D, Giovannucci E, Boffetta P, Fadnes LT, Keum N, Norat T, et al. Fruit and vegetable intake and the risk of cardiovascular disease, total cancer and all-cause mortality-a systematic review and dose-response meta-analysis of prospective studies. Int J Epidemiol. 2017;46(3):1029–56. 28338764 10.1093/ije/dyw319 PMC5837313

[48] Brunault P, Salamé E, Jaafari N, Courtois R, Réveillère C, Silvain C, et al. Why do liver transplant patients so often become obese? The addiction transfer hypothesis. Med Hypotheses. 2015;85(1):68–75. 25896392 10.1016/j.mehy.2015.03.026

[49] Carr MM, Wolkowicz NR, Cave S, Martino S, Masheb R, Midboe AM. Weight change in a National cohort of U.S. Military veterans engaged in medication treatment for opioid use disorder. J Psychiatr Res. 2023;168:204–12. 37918033 10.1016/j.jpsychires.2023.10.012

[50] Ochalek TA, Laurent J, Badger GJ, Sigmon SC. Sucrose subjective response and eating behaviors among individuals with opioid use disorder. Drug Alcohol Depend. 2021;227:109017. 10.1016/j.drugalcdep.2021.109017. Epub 2021 Sep 1. PMID: 34488077. 10.1016/j.drugalcdep.2021.109017 34488077

[51] Chavez MN, Rigg KK. Nutritional implications of opioid use disorder: A guide for drug treatment providers. Psychol Addict Behav. 2020;34(6):699–707. 32202820 10.1037/adb0000575

[52] Meckel KR, Kiraly DD. A potential role for the gut Microbiome in substance use disorders. Psychopharmacology. 2019;236(5):1513–30. 30982128 10.1007/s00213-019-05232-0 PMC6599482

[53] Zhang P. Influence of foods and nutrition on the gut Microbiome and implications for intestinal health. Int J Mol Sci. 2022;23(17). 10.3390/ijms23179588 PMC9455721 36076980

