# Supplementary material for: Experiences with healthy eating among individuals with opioid dependence: insights from a dietary assessment
Source: BMC Nutr. 2025 Jun 5;11:111. doi: 10.1186/s40795-025-01106-1 (PMC12139190; doi:10.1186/s40795-025-01106-1)
Supplement: Supplementary file 1 — Supplementary Material 1 [file 40795_2025_1106_MOESM1_ESM.docx]

**Interview guide; “Barriers and facilitators to healthy eating among individuals with opioid dependence: Insights from a dietary assessment”**

This interview guide has been translated from Norwegian. We confirm that it was developed specifically for this study and it has never been published anywhere else before.

**General information about healthy diet and dietary advice**

- What does a healthy diet consist of for you?
- What significance does food have for you? (source of nourishment, arena for being social, coping with stress, etc.)
  - To what extent do you think diet is important?
  - How do you think that diet affects your physical and mental health?
  - What effect do you think your diet has on how much and which drugs you use?
  - How do you think that your diet can affect the urge/"craving" for drugs?
- Which of the Norwegian dietary guidelines do you know?
- How do you feel that your diet compares to the dietary guidelines that you know?
- Can you describe a time when you were particularly motivated to eat healthier?
  - If yes; What motivated you?
    - What changes were you trying to make? Did they succeed? If they do not succeed; What do you think was the cause?
  - If no; What makes you not feel motivated to change your diet?
    - Do you think your diet is already good enough? Does it feel impractical? Not sure what would be good? Are other areas of life more important? etc
- What makes it more difficult for you to eat healthy?
  - Economy? Practical skills? Stress in everyday life? Dental health? Access to kitchen? Loneliness? Etc

**About the DIGIKOST tool:**

- How did you experience the screening of your diet (with the DigiKost tool)?
  - What did you think was good?
  - What do you think could be improved?
  - How did you experience the questions in the DigiKost questionnaire?
  - Were any of the questions difficult to understand?
- How did you experience the review of the report on your diet (with the DigiKost tool)?
- How do you think the Digi-Kost report matches your diet as you see it?
  - Which part of the results surprised you the most?
- What previous experiences have you had with mapping diet or health digitally?
- What significance do you think the feedback you received about your own diet will have on your choices of food/eating habits in the future?
- What kind of measures can help you to eat healthily? o What kind of support could you have needed to help you with your diet? o In what way can the healthcare system help?
